# The Effect of Wood Aerosols and Bioaerosols on the Respiratory Systems of Wood Manufacturing Industry Workers in Golestan Province

**Published:** 2017

**Authors:** Phateme Badirdast, Mansour Rezazadeh Azari, Soussan Salehpour, Ali Ghadjari, Soheila Khodakarim, Davod Panahi, Moslem Fadaei, Abolfazl Rahimi

**Affiliations:** 1 College of Public Health, Shahid Beheshti University of Medical Sciences, Tehran, Iran; 2 Safety Promotion and Injury Prevention Research Center, College of Public Health, Shahid Beheshti University of Medical Sciences, Tehran, Iran; 3 College of Medicine, Shahid Beheshti University of Medical Sciences, Tehran, Iran; 4 School of Industrial Engineering, Azad University, South Tehran Branch, Tehran, Iran.; 5 HSE Expert in the North Wood Co., Golestan Province, Iran.

**Keywords:** Wood dust, Occupational Exposure, Chipboard, Lung Function

## Abstract

**Background:**

Occupational exposure to dust leads to acute and chronic respiratory diseases, occupational asthma, and depressed lung function. In the light of a lack of comprehensive studies on the exposure of Iranian workers to wood dusts, the objective of this study was to monitor the occupational exposure to wood dust and bioaerosol, and their correlation with the lung function parameters in chipboard manufacturing industry workers.

**Materials and Methods:**

A cross-sectional study was conducted on chipboard workers in Golestan Province; a total of 150 men (100 exposed cases and 50 controls) were assessed. Workers were monitored for inhalable wood dust and lung function parameters, i.e., FVC, FEV_1_, FEV_1_/FVC, and FEF_25–75%_. The workers’ exposure to bioaerosols was measured using a bacterial sampler; a total of 68 area samples were collected. The analysis was performed using the Mann-Whitney, Kruskal-Wallis, and regression statistical tests.

**Results:**

The geometric mean value and geometric standard deviation of inhalable wood dust for the exposed and control groups were 19 ± 2.00 mg/m^3^ and 0.008 ± 0.001 mg/m^3^, respectively. A statistically significant correlation was observed between the lung parameters and cumulative exposure to inhalable wood dust, whereas a statistically significant correlation was not observed between the lung parameters and bioaerosol exposure. However, the exposure of Iranian workers to bioaerosols was higher, compared to their foreign coworkers.

**Conclusion:**

Considering the high level of exposure among workers in this study along with their lung function results, long-term exposure to wood dust may be detrimental to the workers’ health and steps to limit their exposure should be considered seriously.

## INTRODUCTION

Wood dust is produced during the production, processing, and transformation of both hard and soft woods in industries, such as the chipboard production, carpentry, furniture production, and woodcutting industries (1, 2). Widespread use of wood has made it one of the most common occupational exposures in industries (3). The International Agency for Research on Cancer (IARC) has classified hard wood dust as a human carcinogen (4). The Scientific Committee on Occupational Exposure Limit (SCOEL) of the European Union reported that an exposure to wood dust of more than 0.5 mg/m^3^ caused lung symptoms, such as acute or chronic respiratory diseases, occupational asthma, and depression of lung functions (5). Lung function tests of the wood workers demonstrated a remarkable decrease in the mean value of forced vital capacity (FVC), forced expiratory volume during the first second (FEV_1_), and maximum ventilation volume (MVV) (6). A few studies showed a strong statistical correlation between respiratory problems and cumulative exposure to wood dusts (7–9). Occupational exposure to wood dust can be hazardous to the health of the workers. Although wood dust affects all systems of the body, the lungs are more susceptible to airborne pollutants. In the furniture industry, cabinet workshops, and carpentry workshops, symptoms such as cough, fatigue, chest pain, asthma, and headache were reported among the exposed workers (10). Besides wood dust, workers were also exposed to bioaerosols. Oppliger et al. also demonstrated a fungal concentration of more than 3500 colony forming units per cubic meter (CFU/m^3^) at the workplace (11). Moreover, in a study by Sivrikaya and Kara in Turkey, the most common form of fungi in woodworking operations and a major source of respiratory allergy in the workers was the *Penicillium spp*. (12). In the light of the impact of wood dust on the health of the workers, and a lack of prior studies on Iranian wood manufacturing industry workers, the objective of this study was to investigate the occupational exposure to wood dust and bioaerosols, and the lung parameters of chipboard workers in Golestan Province.

## MATERIALS AND METHODS

Cross-sectional, observational and occupational monitoring was carried out on different chipboard manufacturing task groups, such as disembarkation, shredding, fine shredding, milling, drying, adhesive mixing, pressing, sawing, sanding, sanitation, and transportation, were occupationally monitoring in this cross-sectional observational study; the study site was a chipboard manufacturing plant located in Golestan Province. The exposure of the workers to wood dust was personally monitored according to the National Institute of Occupational Safety and Health (NIOSH) Method No. 500 (13). In this method, sampling was performed using an SKC personal sampler and a 25-mm ester cellulose mixed filter with a pore size of 0.8 μm. Before and after sampling, the dried filters were weighed using an analytical balance. In this study, 100 chipboard workers exposed to wood dust, and 50 workers (guards) from the same socioeconomic class without any active exposure to wood dust were monitored for exposure. The lung parameters, such as FVC, FEV_1_, FEV_1_/FVC, and FEF_25–75%_, of all the exposed workers and controls were tested. The lung function tests were performed using a Micro lab II spirometer; with this equipment, all lung function parameters were automatically adjusted for age and BMI. All spirometry tests were performed at the end of their occupational monitoring session for wood dust. Throughout the duration of the spirometry tests, the workers were seated and their noses clamped (14).

The workers were also monitored for their exposure to bioaerosols (15). In this method, area sampling was conducted during the working hours using a bacterial sampler (Casella air bacteria sampler MK II T13962) with a flow of 10 L/min at 1.5 m from the ground, the breathing level for the workers belonging to the 11 task groups (disembarkation, shredding, fine shredding, milling, drying, adhesive mixing, pressing, sawing, sanding, transportation, and sanitation). Sampling was performed on plates containing Sabouraud dextrose agar medium. Subsequently, the samples were sent to a laboratory and incubated at 25°C for 48 hours. All samples were counted in CFU/m^3^ and were investigated under the microscope for identifying the fungus types (16).

Using a questionnaire, demographic data, including height, age, experience, education, and income were collected from all the workers. The inclusion criteria consisted of having at least one year of work experience and being a non-smoker.

The Statistical Package for the Social Sciences (SPSS) version 22.0 for Windows was used for the statistical analyses. The results of the exposure were presented as the geometric mean ± SE. For statistical comparison, a p-value < 0.05 was used as the criterion for statistical significance. One-sample Kolmogorov-Smirnov statistical test was used to determine the normality of the data. Mann-Whitney and Kruskal-Wallis statistical tests were used to analyze the data of the exposed workers and control group. To evaluate the workers’ cross-sectional and cumulative exposure to inhalable wood dust, and their correlation with the lung parameters, linear regression analyses were used.

## RESULTS

The study showed that the mean age and work experience of the exposed workers were 35 years and 6 years, respectively and those for the control group were 34 years and 5 years, respectively (Table 1).

**Table 1.** Demographic data of workers

**Characteristics**	**Sample (n=100)**		**Control (n=50)**	

	SD	Mean	SD	Mean
**Age (years)**	2.26	35	2.28	34
**Experience (years)**	3.46	6	3.19	5

**Figure 1.**
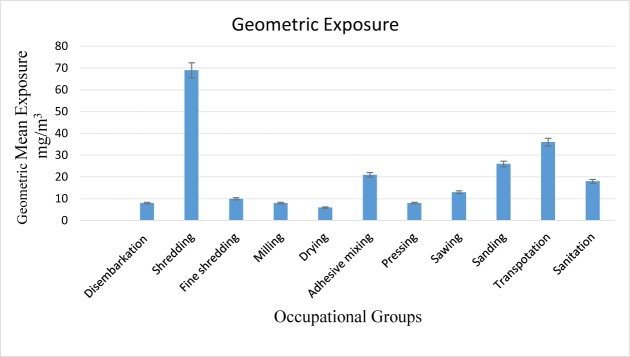
The geometric mean of exposure of different task groups to inhalable dust as mg/m^3^

Inhalable wood dust exposures of the exposed and control groups followed a normal distribution, according to the Kolmogorov-Smirnov test (p-value=0.066). The geometric mean value and geometric standard deviation of inhalable wood dust for the exposed and control groups were 19 ± 2.00 mg/m^3^ and 0.008 ± 0.001 mg/m^3^, respectively. The workers’ exposure to inhalable wood dust in the exposed groups had statistically significant differences (p-value<0.0001; Figure 1). The highest exposure to inhalable wood dust was noted for the shredding operation.

The lung function parameters, FVC, FEV_1_, FEV_1_/FVC, and FEF_25–75%_, followed a normal distribution according to the Kolmogorov-Smirnov test (p-value>0.05). Results pertaining to the lung function of workers in the exposed and control groups are displayed in Table 2. The lung function parameters FEV_1_ and FVC of the exposed group were statistically significantly lower than those of the control group (p-value<0.0001). Moreover, the lung function parameter FEV_1_/FVC in the exposed group was statistically significantly higher than that in the control group (p-value<0.0001). There was no statistically significant difference in the lung function parameter FEF_25–75%_ between the exposed and control groups (p-value = 0.55; Table 2).

**Table 2.** Lung function parameters of exposed and control workers

**Respiratory Parameters (Land%)**	**Exposed (n=100)**		**Control (n=50)**		**P-value**

	**Mean**	**SE**	**Mean**	**SE**	
**FVC(L)**	3.90	0.005	4.85	0.06	0.0001
**FEV1(L)**	3.52	0.008	4.01	0.06	0.0001
**FEV_1_/ FVC (%)**	90.51	0.08	80.62	0.21	0.0001
**FEF_25–75%_(L/S)**	4.47	0.01	4.48	0.06	0.55

**Figure 2.**
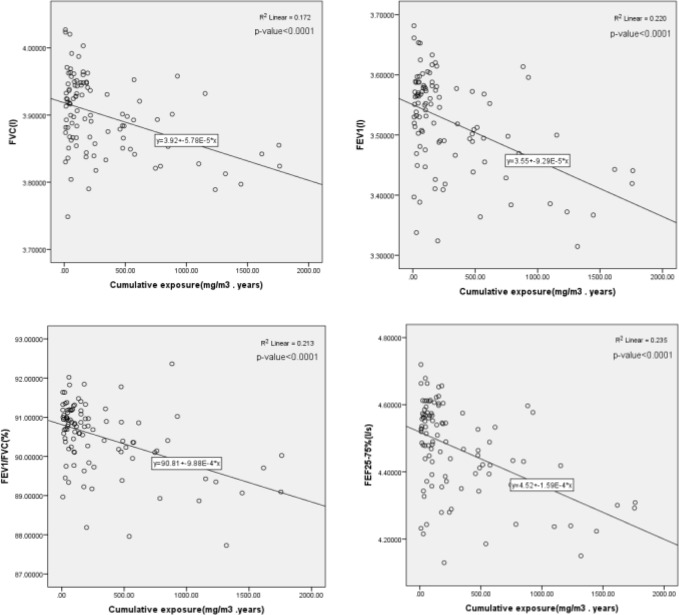
Regression of lung parameters and cumulative exposure to inhalable wood dust

To study the relationship between the lung parameters FVC, FEV_1_, FEV_1_/FVC, and FEF_25–75%_, and the level of exposure to inhalable wood dust, the lung parameters were adjusted for age, height, and weight. No meaningful correlation was observed between the cross-sectional exposure to inhalable wood dust and decrease in the lung parameters FVC, FEV_1_, FEV_1_/FVC, and FEF_25–75%_ on regression analysis (Table 2). However, a similar regression analysis of the cumulative exposure (multiplication of the typical exposure and individual work history) to inhalable dust and the lung function parameters FVC, FEV_1_, FEV_1_/FVC, and FEF_25–75%_ showed a meaningful correlation (p-value > 0.0001) (Figure 2).

**Figure 3.**
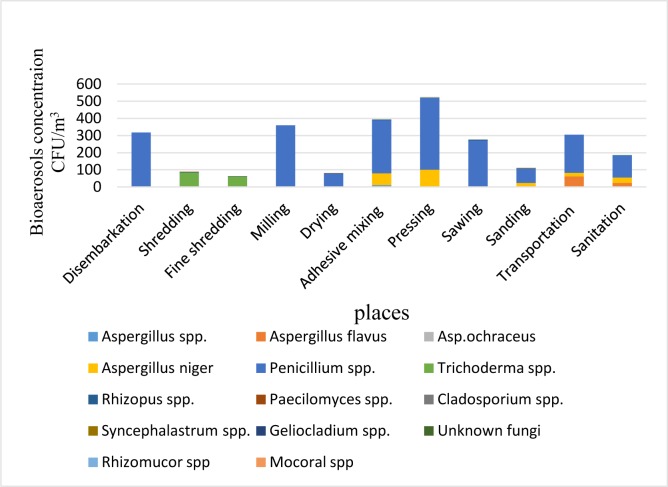
Concentration of bio-aerosols (CFU/m^3^) for chipboard task groups

The mean value of the concentration of bioaerosols in the workplace was 269 CFU/m^3^. Fungal species, namely, *Penicillium spp. Aspergillus niger*, *Asp. ochraceus*, *Asp. flavus*, *Trichoderma spp., Rhizopus spp., Aspergillus spp., Cladosporium spp*., Mucorales, *Rhizomucor spp., Syncephalastrum spp., Paecilomyces spp*., *Geliocladium spp.,* and unknown fungi, were found in the samples. The most abundant fungi in the samples were either *Penicillium spp.* or *Aspergillus niger.* Bacteria were also found in a limited number of samples (Figure 3).

## DISCUSSION

In this study, the workers were monitored for occupational exposure to inhalable wood dust and bioaerosols, and their correlation with the lung function test results was evaluated. The exposed workers were divided into 11 task groups according to the workers’ job descriptions. The shredding and transportation groups had the highest exposures to inhalable wood dust. Considering that the type of wood used in the chipboard factories was not a specific type of wood, and due to the use of wood waste from other plants, the threshold limit value (TLV) for non-carcinogenic wood dust was set at 1 mg/m^3^, according to the American Conference of Governmental Industrial Hygienists guidelines; this was considered in the risk evaluation of chipboard workers (17). The occupational exposure of 98% of the chipboard factory workers was several times higher than the TLV set for the least harmful type of wood dust. Comparing the exposure of chipboard workers in Golestan Province with their coworkers abroad, the Iranian workers’ exposure to inhalable wood dust was 20–80 times higher than that of their foreign counterparts (18–23). The high levels of exposure to wood dust and bioaerosols in a chipboard plant in Golestan might be due to its primitive processing, inappropriate conditions of wood storage, and lack of engineering control measures for wood dust.

Moreover, the reduction in lung capacity parameters (FVC, FEV_1_, FEV_1_/FVC, and FEF_25–75%_) of workers in the wood industry and the significant negative statistical correlation between the cumulative exposures to wood dust and lung function parameters observed in this study are in line with the findings of other similar studies (24–27). Interpretation of the lung function tests of all exposed wood workers and the spirometry tests in this study were performed according to the criteria proposed by Johnson and Theurer (28). In this study, 52% of the exposed workers had normal respiratory functions, while 33% demonstrated restrictive and 15% obstructive lung function complications. The results of this study were in line with the recent studies showing a higher percentage of restrictive and obstructive lung conditions in exposed wood workers than in the control populations (27, 29–31). However, other studies have expressed doubts regarding the detrimental effects of wood dust on the pulmonary system (32, 33). The former assumption raises some questions, since even low exposure of workers to wood dust could occur simultaneously with exposure to other pollutants, such as formaldehyde (34, 35), glue and resin (36), solvents (37), silica (38), and bioaerosols (39). Therefore, the author of this study believes that restrictive and obstructive lung complications in wood manufacturing workers cannot not be ruled out.

According to the airborne bioaerosol results of this study, *Penicillium spp.* fungus was found to be the most common type affecting chipboard workers. The levels of Iranian chipboard workers’ exposure to bioaerosols were much higher than those of their foreign coworkers. Despite Iranian chipboard workers’ exposure to high levels of airborne bioaerosols, a statistically significant relationship was not observed between the exposure to bioaerosols and lung function parameters. Other authors have also stated that due to the uncertainties regarding the assessment of workers’ exposure to bioaerosols through culture quantifications, definitive conclusions about its effects on the lung function could not be reached; hence, an alternative method for bioaerosol monitoring was recommended (40). Despite the doubts about bioaerosol monitoring via quantification in terms of CFU/m^3^ and the lack of an appropriate standard for airborne biological agents in the workplace, the existence of *Penicillium* fungus in the air at the workplace might be responsible for respiratory system complications, such as allergic rhinitis (41).

This is the first report on the risk evaluation of Iranian wood workers to airborne wood dust and bioaerosols, and their correlation with respiratory lung function parameters. Based on the data provided by this study on the excessive exposure to wood dusts and the statistically significant correlation between cumulative exposure and depression of lung function parameters and pulmonary status, appropriate risk management of exposed workers is recommended.
